# Metabolic Composition of Methanolic Extract of the Balkan Endemic Species *Micromeria frivaldszkyana (Degen)* Velen and Its Anti-Inflammatory Effect on Male Wistar Rats

**DOI:** 10.3390/ijms25105396

**Published:** 2024-05-15

**Authors:** Kristina Stavrakeva, Kalina Metodieva, Maria Benina, Anelia Bivolarska, Ivica Dimov, Mariya Choneva, Vesela Kokova, Saleh Alseekh, Valentina Ivanova, Emil Vatov, Tsanko Gechev, Tsvetelina Mladenova, Rumen Mladenov, Krasimir Todorov, Plamen Stoyanov, Donika Gyuzeleva, Mihaela Popova, Elisaveta Apostolova

**Affiliations:** 1 Department of Pharmacology, Toxicology, and Pharmacotherapy, Faculty of Pharmacy, Medical University of Plovdiv, Vasil Aprilov Str. 15A, 4002 Plovdiv, Bulgaria; kristina.stavrakeva@mu-plovdiv.bg (K.S.); vesela.kokova@mu-plovdiv.bg (V.K.); 2 Department of Medical Biochemistry, Faculty of Pharmacy, Medical University of Plovdiv, Vasil Aprilov Str. 15A, 4002 Plovdiv, Bulgaria; kalina.metodieva@mu-plovdiv.bg (K.M.); anelia.bivolarska@mu-plovdiv.bg (A.B.); ivica.dimov@mu-plovdiv.bg (I.D.); mariya.choneva@mu-plovdiv.bg (M.C.); 3 Center of Plant Systems Biology and Biotechnology, 14, Sveti Knyaz Boris I Pokrastitel, Str., 4023 Plovdiv, Bulgaria; benina@cpsbb.eu (M.B.); alseekh@cpsbb.eu (S.A.); vivanova@cpsbb.eu (V.I.); vatov@cpsbb.eu (E.V.); gechev@cpsbb.eu (T.G.); 4 Max Planck Institute of Molecular Plant Physiology, 1 Am Muehlenberg, 14476 Potsdam, Germany; 5 Department of Botany and Biological Education, Faculty of Biology, University of Plovdiv “Paisii Hilendarski”, 24 Tsar Assen Str., 4000 Plovdiv, Bulgaria; cmladenova@uni-plovdiv.bg (T.M.); rummlad@uni-plovdiv.bg (R.M.); ktodorov@uni-plovdiv.bg (K.T.); pstoyanov@uni-plovdiv.bg (P.S.); dgyuzeleva@uni-plovdiv.bg (D.G.); 6 Department of Bioorganic Chemistry, Faculty of Pharmacy, Medical University of Plovdiv, Vasil Aprilov Str. 15A, 4002 Plovdiv, Bulgaria; 7 Faculty of Pharmacy, Medical University of Plovdiv, Vasil Aprilov Str. 15A, 4002 Plovdiv, Bulgaria; ff1976@mu-plovdiv.bg; 8 Research Institute, Medical University of Plovdiv, 4002 Plovdiv, Bulgaria

**Keywords:** *Micromeria frivaldszkyana*, methanolic extract, GC-MS, UPLC-MS-MS, plant metabolites, mineral content, acute toxicity, anti-inflammatory effect, rat paw edema, carrageenan

## Abstract

Extracts from medicinal plants are widely used in the treatment and prevention of different diseases. *Micromeria frivaldszkyana* is a Balkan endemic species with reported antioxidant and antimicrobial characteristics; however, its phytochemical composition is not well defined. Here, we examined the metabolome of *M. frivaldszkyana* by chromatography–mass spectrometry (GC-MS), ultra-performance liquid chromatography–mass spectrometry (UPLC-MS-MS), and inductively coupled plasma mass spectrometry (ICP-MS). Amino acids, organic acids, sugars, and sugar alcohols were the primary metabolites with the highest levels in the plant extract. Detailed analysis of the sugar content identified high levels of sucrose, glucose, mannose, and fructose. Lipids are primary plant metabolites, and the analysis revealed triacylglycerols as the most abundant lipid group. Potassium (K), magnesium (Mg), zinc (Zn), and calcium (Ca) were the elements with the highest content. The results showed linarin, 3-caffeoil-quinic acid, and rosmarinic acid, as well as a number of polyphenols, as the most abundant secondary metabolites. Among the flavonoids and polyphenols with a high presence were eupatorin, kaempferol, and apigenin—compounds widely known for their bioactive properties. Further, the acute toxicity and potential anti-inflammatory activity of the methanolic extract were evaluated in Wistar rats. No toxic effects were registered after a single oral application of the extract in doses of between 200 and 5000 mg/kg bw. A fourteen-day pre-treatment with methanolic extract of *M. frivaldszkyana* in doses of 250, 400, and 500 mg/kg bw induced anti-inflammatory activity in the 1st, 2nd, and 3rd hours after carrageenan injection in a model of rat paw edema. This effect was also present in the 4th hour only in the group treated with a dose of 500 mg/kg. In conclusion, *M. frivaldszkyana* extract is particularly rich in linarin, rosmarinic acid, and flavonoids (eupatorin, kaempferol, and apigenin). Its methanolic extract induced no toxicity in male Wistar rats after oral application in doses of up to 5000 mg/kg bw. Additionally, treatment with the methanolic extract for 14 days revealed anti-inflammatory potential in a model of rat paw edema on the 1st, 2nd, and 3rd hours after the carrageenan injection. These results show the anti-inflammatory potential of the plant, which might be considered for further exploration and eventual application as a phytotherapeutic agent.

## 1. Introduction

Plants are capable of synthesizing different phytochemicals, some of which are specific for the genus or the family. Currently, interest in the biological effects of plants is increasing [1]. However, the general interest is focused on plants that are well known in traditional phytomedicine like *Rosmarinus officinalis*, *Lavandula officinalis*, *Curcuma longa*, *Berberis vulgaris*, etc. Many endemic plants remain out of the scope of scientific studies, and information about their phytochemical composition and biological effects is scarce [2].

**Figure 1:**
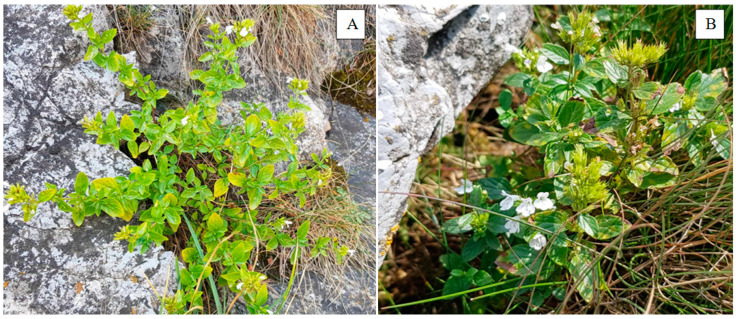
*M. frivaldszkyana*. (**A**) General view in its natural habitat; (**B**) general view with flowers.

*Micromeria frivaldszkyana* (*M. frivaldszkyana*) is a species endemic to Bulgaria (Figure 1). It is included in the Biological Diversity Act (Appendix 3), and the Red Data Book of the Republic of Bulgaria classifies it as “endangered” [3,4,5]. 

The phytochemical composition of *M. frivaldszkyana* was initially reported by Vukelić [6] and Nikolova et al. [7]. Thin-layer chromatography of a methanolic extract of the plant revealed the presence of rutin, quercetin, naringin, kaempferol 3-rutinoside, chlorogenic acid, and rosmarinic acid (RA). Recently, the phenolic components in the aerial parts of *M. frivaldszkyana* were identified, with RA being the most abundant compound. The flavonoids hesperidin, epicatechin, quercetin, kaempherol, and apigenin were also present in high amounts in the plant [2]. 

Plants from the genus *Micromeria* are used in the ethnopharmacology for the treatment of cardiac diseases, pulmonary pathologies (asthma), and as a wound healing remedy. Other indications include the treatment of headache, cold, infections of the skin, etc. Scientific reports have revealed antirheumatic, antiseptic, and central nervous system (CNS)-stimulating properties of different species of this genus; however, the information about *M. frivaldszkyana* is scarce [7,8,9,10]. Significant anti-inflammatory, hepato-, and gastroprotective activities have been reported for *Micromeria fruticosa* [11]. The anti-inflammatory effect of *M. fruticosa* has been attributed to the high content of rutin, apigenin, tectochrysin, and glucosides such as 7,4’ dihydroxyflavone-7-rhamnoglucoside, all of which exhibit a high potential for inhibition of the NF-kB pathway [12]. A study on *Micromeria biflora* has revealed anti-inflammatory, analgesic, and antipyretic properties, which are likely due to the high concentration of caryophyllene oxide and β-eudesmol detected in the plant’s essential oil [13].

Currently, the only data available in the scientific literature regarding the biological effects of *M. frivaldszkyana* extracts are reports on the antimicrobial and antioxidant activities of the plant, accompanied with some information about its phytochemical metabolites [2,7]. Similar effects are reported for plants of the genus *Stachys*, which also belong to the *Lamiaceae* family [14]. No data on the toxicity of the methanolic plant extract or its effects on the inflammation processes were found.

**Figure 2:**
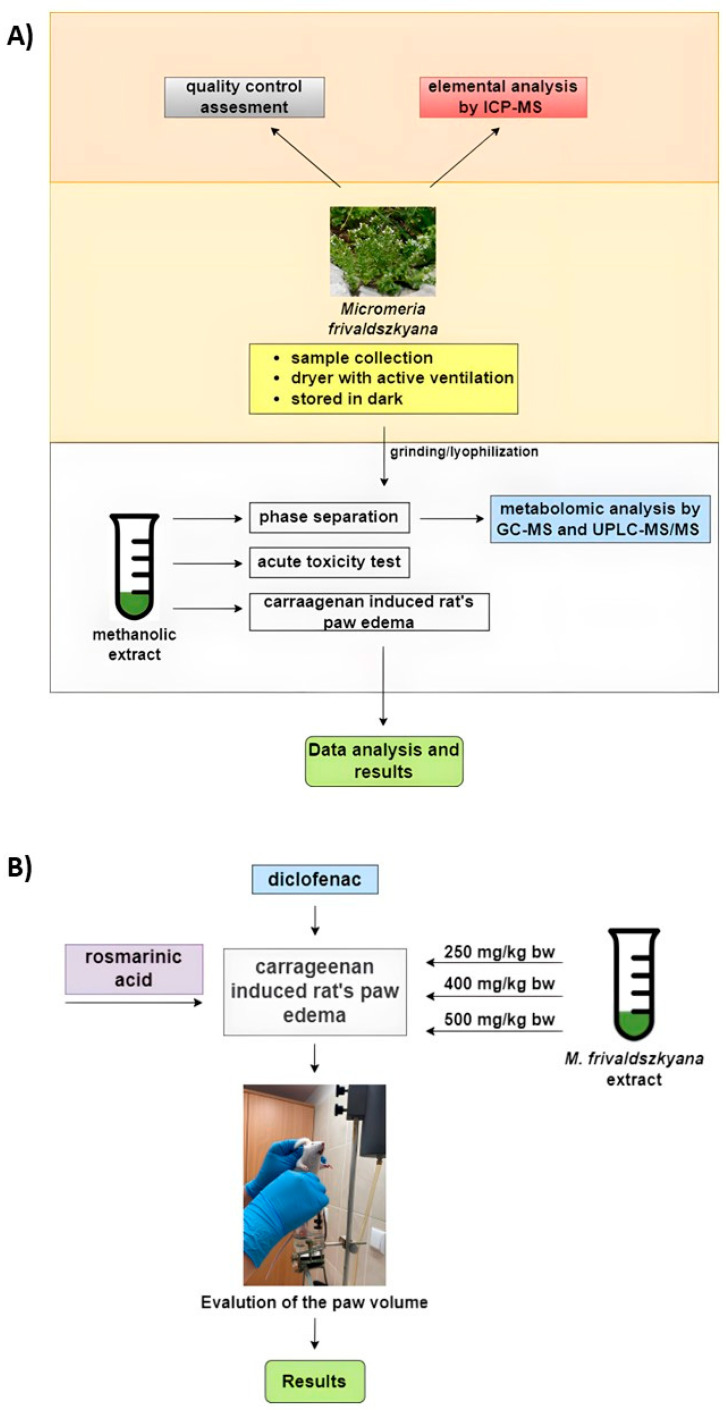
Schematic illustration of the experimental design. (**A**). General overview; (**B**). Anti-inflammatory evaluation of *M. frivaldszkyana* methanolic extract in vivo.

Given the little data on the species *M. frivaldszkyana*, we decided to evaluate its therapeutic potential. Previously, Mladenova et al. [2] reported the organic acid content, the content of sugars, and the polyphenol composition in the aerial parts of the plant. In our study, we aimed to complete a phytochemical analysis and detect some biological effects of the methanolic extract of the plant. The workflow task assignment and study design (Figure 2A) consisted of: (1) an evaluation of the phytochemical composition of the methanolic extract of the plant, as well as a quality assessment and mineral content analysis of *M. frivaldszkyana* herba; (2) an acute toxicity study in rats; and (3) a 14-day oral application in rats followed by carrageenan-induced paw edema (Figure 2B).

## 2. Results

*M. frivaldszykyana* lyophilized dry powder was subjected to metabolite extraction by using 70% methanol and a subsequent fractionation. The polar phase was used for both the GC-MS and UPLC-MS-MS analyses, and the non-polar fraction was used for the lipid investigation. 

### 2.1. GC-MS Analysis of Primary Metabolites

**Figure 3:**
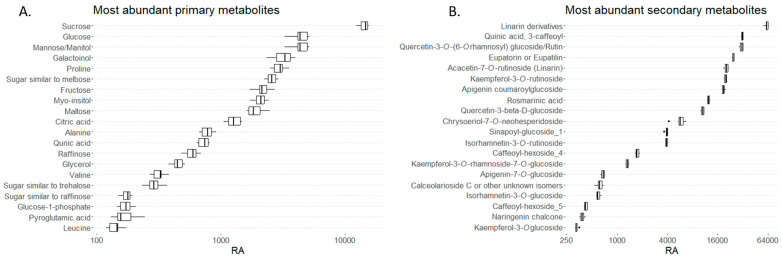
(**A**) A boxplot representing primary metabolites in *M. frivaldszkyana* ordered according to their relative abundance (RA) (x-axes). The presented data are from six biological replicates measured by GC-MS. Black lines within the boxes indicate medians, and whiskers indicate upper and lower quantiles. (**B**) Boxplots of secondary metabolites in *M. frivaldszkyana* measured by UPLC-MS-MS and ordered according to their relative abundance (RA) (*n* = 6). Black lines within the boxes indicate medians, whiskers indicate upper and lower quantiles, and black dots indicate outliers.

The GC-MS analysis resulted in the identification of 83 compounds, classified as amino acids, organic acids, sugars, and sugar alcohols (Table S1 Supplementary Material). Sucrose was the most abundant metabolite, followed by different sugars and sugar alcohols such as glucose, mannose, fructose, maltose, galactoinol, myo-inositol, and glycerol (Figure 3). In addition, significant amounts of citric and quinic acids were detected. Proline exhibited the highest levels among the amino acids, followed by alanine. Relatively high amounts of valine and pyroglutamic acid were also detected in the samples. Overall, the analysis of the semi-polar phase showed a high presence of sugars and sugar alcohols. 

**Figure 4:**
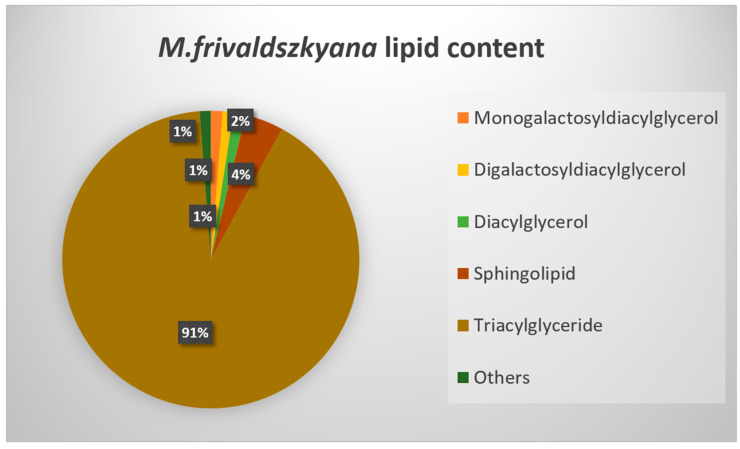
Lipid content of *M. frivaldszkyana*. Lysophospholipids, lysomonogalactosyldiacylglycerols, lysodigalactosylglycerols, phospholipids, phosphatidylcholine, and phosphatisylethanolamine are given as “Others”, since they represent less than 1% of the total lipid amount.

Lipids are primary plant metabolites; therefore, a lipidomic study on the non-polar fraction was also conducted. A total of 163 lipid compounds were identified and distributed in 10 lipid classes: diacylglycerols, digalactosyldiacylglycerols, lysophospholipids, lysomonogalactosyldiacylglycerols, lysodigalactosyldiacylglycerols, monogalactosyldiacylglycerols, phosphatidylcholine, phospholipids, sphingolipids, and triacylglycerols (Figure 4). In order to better visualize the lipid type distribution, the respective percentages of the total lipid content for each lipid group are presented. The results showed triacylglycerols as the most abundant lipid group. 

### 2.2. UPLC-MS/MS Analysis of Secondary Metabolites

The untargeted UPLC-MS-MS analysis of the *M. frivaldszkyana* methanolic extract samples resulted in the detection of 192 compounds. The number of the identified compounds was 123, while 69 remain unknown (Table S1 Supplementary Material). The secondary metabolites with highest concentrations were flavonoids, predominantly flavonoid glucosides. The highest levels in the samples were registered for linarin and its derivatives, quinic acid, and derivatives of quercetin, kaempferol, naringenin, and apigenin. Rosmarinic acid was among the most prominent substances detected and was further selected as a reference compound in the experimental design (Figure 3B). 

### 2.3. Quality Control Assesment of M. frivaldszkyana Herba

The quality control assessment of the plant material included a determination of the dry matter moisture content (TH), pH, and ash and organic matter content as well as elemental analysis by ICP-MS. The results are presented in Table 1 and Table 2. As evident in Table 1, the TH of the dried *M. frivaldszkyana* plants was found to be 19.4 ± 0.92, indicating a relatively high water content. The pH of the obtained filtrate was slightly acidic, with values ranging around 5.853 ± 0.032. The percentage of ash after the volatilization of the organic matter was found to be 11.72 ± 0.043. 

As presented in Table 2, twelve of the initial nineteen elements that we screened for were detected, and the respective results are displayed. Potassium (K), magnesium (Mg), zinc (Zn), and calcium (Ca) were the most abundant metal constituents. It is noteworthy that the heavy metals cadmium (Cd), chromium (Cr), cobalt (Co), and lead (Pb) were below the limit of detection. 

### 2.4. Acute Toxicity of Methanolic Extract of M. frivaldszkyana

During the evaluation of the acute toxicity, no toxic effects were registered after a single oral application of the extract in doses ranging from 200 to 5000 mg/kg bw, as shown in Table 3. Based on these results, we estimated the doses for further evaluation of the effects of the extract as 250 and 500 mg/kg bw. According to Hanafy et al. [15], the doses suitable for further evaluation of the pharmacological effects of extracts of natural origin are 1/10 and 1/20 of the estimated LD50. We added the intermediate dose 400 mg/kg to obtain more detailed data on the potential effect of the extract.

### 2.5. Effect of M. frivaldszkyana Methanolic Extract on Carrageenan-Induced Rat Paw Edema 

**Figure 5:**
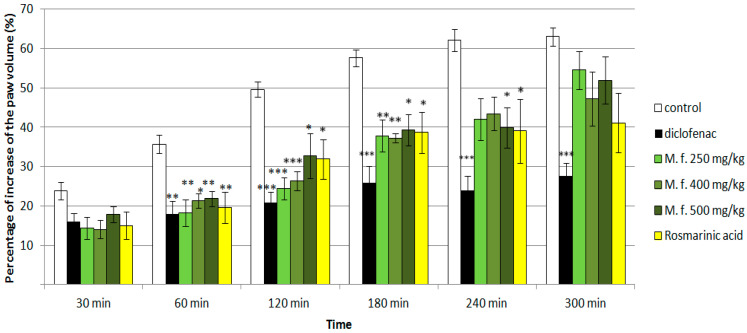
Effects of diclofenac (25 mg/kg bw), *M. frivaldszkyana (M. f)* methanolic extract in three doses (250 mg/kg bw, 400 mg/kg bw, and 500 mg/kg bw), and rosmarinic acid (30 mg/kg bw) on paw edema induced by carrageenan in rats. * *p* < 0.05 vs controls at the same hour; ** *p* < 0.01 vs controls at the same hour; *** *p* < 0.001 vs controls at the same hour.

No statistical significance was registered 30 min after the application of the carrageenan. The standard anti-inflammatory drug diclofenac decreased the paw edema at all the tested time points (60th to 300th minute of the experiment). The methanolic extract of *M. frivaldszkiana* in all the tested doses (250 mg/kg bw, 400 mg/kg bw, and 500 mg/kg bw) showed a well-defined anti-inflammatory effect at the 1st, 2nd, and 3rd hours after the application when compared to the controls. The effect was also present at the 4th hour, but only for the highest tested dose of 500 mg/kg bw. Similar results were obtained for RA, which exhibited anti-inflammatory activity at the same time points (1st, 2nd, 3rd, and 4th hours after the injection) in comparisson to the control rats (Figure 5).

## 3. Discussion

Plants synthesize a huge variety of secondary metabolites and could be used as sustainable sources of phytochemicals with potential biological activity [16]. *Micromeria frivaldszkyana* is an endemic Bulgarian species, the biochemical content and therapeutic activities of which remain unknown. The literature overview revealed a few studies that aimed investigating the main compounds in the plant’s aerial parts or evaluating its antioxidant and antimicrobial effects [2,17].

The literature reports very scarce information about the phytochemical composition of *M. frivaldszkyana*; therefore we decided to study its metabolome as an attempt to reveal the main natural substances that contribute to its pharmacological activities by the application of several analytical platforms, namely, GC-MS, UPLC-MS-MS, and ICP-MS. Gas chromatography–mass spectrometry (GC-MS) is an excellent approach for metabolomic studies in terms of robustness and reproducibility due to its efficiency, selective nature, and database availability [18]. It is widely used in the evaluation of low-molecular-weight compounds, mostly primary metabolites [19]. Generally, primary metabolites are vital for plant growth and development but have no relation to plants’ biological activities. Here, we decided to explore the primary metabolome due to its relation to secondary biochemical pathways, which will provide a better understanding of the plant’s specific metabolism as the natural machinery of its bioactive agents. In addition, a lipidomic analysis was performed, as recent studies have revealed pharmacological activities of some members of this class of phytochemicals [20]. Further, a high-throughput UPLC-MS-MS untargeted screening was applied in order to define the main groups of secondary substances and to identify the most important compounds that may play a significant role in the plant’s therapeutic properties. 

Finally, an ICP-MS approach aimed to complete the whole dataset and to give additional value to the biochemical content of *M. frivaldszkyana’s* metabolome, especially when used as a nutritional supplement [21]. The results obtained in our study seem to differ from those of other species of the same genus that found a significantly higher content of Ca (13,223 mg/kg) and Na 109 mg/kg) [22], probably due to the influence of environmental conditions. The content of other elements (K, Zn, Mn) was significantly higher in the *M. frivaldszkyana* samples compared to *M. croatica* and *M. pseudocroatica.* [23]. The quality control assessment of the dried plant material revealed that the moisture content, pH value, and the present heavy metals were within the permitted range of FAO/WHO regulations. 

The present work aimed to reveal *M. frivaldszkyana’s* phytochemical composition and its bioactive properties. The results from the conducted metabolomic research confirmed a significant part of the already published data in terms of the sugar and organic acid content [2] but added additional value in terms of the polyphenol composition. Linarin and acacetin 7-*O*-rutinoside and its derivatives were found to be among the most abundant secondary metabolites, together with RA and several other flavonoid glycosides. Flavonoids and their glucosides are reported as compounds with different biological activities and low toxicity [24].

According to the authors’ knowledge, this is the first study on the toxicity of *M. frivaldszkyana* extract in vivo. The results showed no toxicity after the oral application of a methanolic extract of the plant in male Wistar rats. However, additional experiments are needed in order to extrapolate these results to other biological species.

Carrageenan-induced paw inflammation in rats is a model of inflammation that is commonly used in the research of new anti-inflammatory agents. This model was chosen for our research due to the possibility of evaluating the specific phase of inflammation in which the extract is active. The inflammatory response to carrageenan consists of two phases. During the first phase (1 h after the injection) the observed inflammation is related to the increased production of histamine, serotonin, and bradykinin, whereas the second phase is governed by the production of neutrophil-derived free radicals, pro-inflammatory cytokines, NO synthesis, neutrophil infiltration, and COX-2 activation and subsequently prostaglandin production [25,26]. Based on the registered activity in a specific phase, we could predict the possible target molecules to which this activity is related.

Our results indicated that *M. frivaldszkyana* methanolic extract applied orally for 14 days alleviated the paw edema during the early phase of inflammation (1st to 3rd hours after carrageenan injection). This result could be related to decreased free-radical production, decreased levels of pro-inflammatory cytokines, NO, or reduced COX-2 activity. Previously, the antioxidant activity of *M. frivaldszkyana* methanolic extract in vitro was reported [7]. Moreover, the *M. frivaldszkyana* extract demonstrated the highest antioxidant activity in comparison to the other studied *Micromeria* species (*M. dalmatica*, *M. juliana*, and *M. cristata*) [7]. A strong antioxidant activity was also registered for ethanolic extracts from the plant, probably due to the high concentrations of phenolic acids (9004 mg/100 g) and flavonoids (594.2 mg/100 g) in the aerial parts of the plant [2]. Based on these reports, we can speculate that the registered anti-inflammatory activity could be related to the antioxidant properties of the extract. However, anti-inflammatory activity is also reported for some of the compounds that were detected in high percentages in the methanolic extract. For example, anti-inflammatory properties (decreased COX-2 activity and NO production) are reported for linarin in vitro [27,28,29], while in vivo experiments showed decreased TNF-α levels in the serum of LPS-challenged mice [28]. However, in our experiment, we did not include linarin as a standard due to its low absorption after oral administration (0.47% bioavailability) [30].

The second most abundant metabolite in the extract was 3-*O*-caffeoylquinic (chlorogenic acid), which is also well known for its anti-inflammatory activity. In vitro experiments demonstrated decreased NO production and pro-inflammatory cytokine (IL-1β, IL-6, TNF-α) levels in LPS-challenged RAW 264.7 macrophages [31]. Oral administration of the compound activated the Keap1/Nrf2 pathway and suppressed inflammation in thioacetamide-challenged rats [32,33]. Chlorogenic acids also interfere with the Nf-kB pathway and induce changes in the levels of pro- and anti-inflammatory cytokines [34,35]. Similarly, anti-inflammatory activity is reported for rutin and its glycoside in vitro [36]. The proposed mechanisms include decreased levels of IL-6, IL-1β, and TNF-α and suppression of the Nf-kB/MAPK pathway [37]. Moreover, this compound decreased the edema in the same model of paw inflammation in rats [38].

Anti-inflammatory effects were also reported for eupatorin in carrageenan-induced paw edema in mice and a model of ear inflammation induced by 12-O-tetradecanoylforbol-13-acetate. The possible mechanism is related to suppression of the synthesis of TNF-α and COX-2 [39,40,41].

The literature overview revealed studies on the anti-inflammatory activity of kaempferol and apigenin, compounds that also had a high presence in the methanolic extract. Reports on their activity in vitro and in vivo are summarized in many review articles, some of which also include results from clinical trials [42,43,44,45].

The compound with the most well-known anti-inflammatory activity, which was also abundant in the extract, is RA. The anti-inflammatory activity of RA is well established; moreover, there are several reports on its activity in carrageenan-induced paw edema [46,47,48].

We can conclude that the registered anti-inflammatory activity of the extract is due to the high content of flavonoids, for which such properties are reported (chlorogenic acid, rutin, eupatorin, kaempferol, apigenin, RA, etc.).

## 4. Materials and Methods

### 4.1. Plant Material and Methanolic Extract Preparation

Aerial parts of *M. frivaldszkyana* were collected during the vegetation period of 2019–2020 within the Bulgarka Nature Park, floristic region of Middle Stara Planina Mountain. Herbarium samples from *M. frivaldszkyana* were deposited in the herbarium of the Agricultural University, Plovdiv (SOA), with the designated reference number 062648. The plant material was dried naturally in a shaded area at room temperature and was finely ground using a mechanical grinder until reaching a powder size of less than 400 μm. The ground samples were stored in paper bags until the analysis was conducted.

For the extraction process, 10 g of powdered plant material was extracted in 70% methanol (1:10 *w*/*v*). The mixture was continuously stirred for 24 h at room temperature in a flask protected from light. In addition, during this period, a triplicate ultrasonic extraction was performed, consisting of 3 cycles of 15 min at 30 °C. After centrifugation at 6000 g for 15 min, the resulting supernatant was filtered using Whatman No. 1 filter paper. The same extraction procedure was repeated twice on the remaining plant material. The three extracts were combined, and the solvent was evaporated under reduced pressure using a rotary evaporator (Heidoplh, Germany) at 50 °C until complete dryness was achieved. 

### 4.2. Metabolite Extraction and Metabolite Measurements 

Metabolite extraction was implemented as reported by Giavalisco et al. [49] and Salem et al. [50] with some modifications. 

Approximately 15 mg of lyophilized plant material was extracted by adding 70% methanol. The samples were vortexed for 10 min at 4 °C and sonicated for 10 min in a sonication bath with ice, followed by centrifuging for 10 min. The obtained methanolic extract was then filtered.

The primary metabolites were analyzed by GC-MS with an obligatory step of derivatization, performed according to Lisec et al. [51]. Briefly, 40 μL of 20 mg ml^−1^ methoxyamine hydrochloride (cat. no. 593-56-6, Sigma, Burlington, MA, USA) in pyridine (cat. no. 110-86-1, Merck, Rahway, NJ, USA) was added to the vacuum-dried methanol extracts and incubated at 37 °C for 120 min, followed by another 30 min treatment at 37 °C with 70 μL of trimethylsilyl-N-methyl trifluoroacetamide (MSTFA, Ref. no. 701270.510, Macherey-Nagel, Düren, Germany). A total of 1 µL of the samples was injected by an autosampler (Gerstel Multi-Purpose system, Gerstel GmbH & Co.KG, Mülheim an der Ruhr, Germany) in the splitless mode to a chromatograph coupled to a time-of-flight mass spectrometer system (Leco Pegasus HT TOF-MS; LECO Corporation, St. Joseph, MI, USA). The carrier gas was helium with a constant flow rate of 2 mL s^−1^. Gas chromatography was performed on a DB-35 column (capillary column, 30 m length, 0.32 mm inner diameter, 0.25 μm film thickness, PN: G42, Agilent, Santa Clara, CA, USA). The injection temperature was 230 °C; the transfer line and ion source were set to 250 °C. The temperature of the oven was initially set at 85 °C and increased at a rate of 15 °C.min-1 up to a final temperature of 360 °C. After a solvent delay of 180 sec, mass spectra were recorded at 20 scans s-1 with a 70–600 *m*/*z* scanning range.

For the specialized metabolites, the aqueous methanol extracts were transferred to LC-MS glass vials and analyzed using Thermo Q Exactive Focus (Thermo Scientific, Waltham, MA, USA) with a reverse-phase C18 column held at 40 °C with a flow rate of 400 µL/min and with gradual changes in eluent A, water, and B, acetonitrile, both stabilized with 0.1% formic acid. Mass spectra were acquired in full-scan MS2, with the high-energy collisional dissociation (HCD) energy set at 30eV in both the positive and negative modes.

For lipid analysis, 15 mg homogenized plant tissues were carefully vortexed for 1 min in the presence of 1 -mL of pre-cooled (−20 °C) methyl tert-butyl ether/methanol (3:1 *v*/*v*). Afterwards, the tubes were placed on an orbital shaker (1000 rpm) for 15 min at 4 °C, followed by 15 min of sonication. A total of 500 µL of a water/methanol solution (3:1 *v*/*v*) was used for phase separation. The tubes were vortexed again for 1 min and further centrifuged at 11,200 rpm for 5 min. A total of 400 µL of the upper lipophilic phase was dried, re-suspended in an actontrile/isopropanol mixture (7:3) and analyzed on an Orbitrap LC-MS system (Exactive, Thermo Scientifc). A reverse-phase C8 column at 60 °C running with a flow of 400 µL/min was used, and the mass spectra were acquired in the full-scan MS in the positive ionization mode with a mass range of 150–1500 *m*/*z*.

### 4.3. Compound Annotation

The annotation of the metabolites analyzed by GC-MS was carried out using the Golm Metabolome Database [52]. Lipid annotation was performed by an in-house library search based on the full-scan MS^1^, consisting of standalone standards as described in Hummel et al. [53].

### 4.4. Quality Control Assesment of M. frivaldszkyana Tissue

#### 4.4.1. Dry Matter Moisture Content 

The analysis was performed in accordance with the guidelines of the French association of standardization standard (NF-V03-402, 1985) [54] by placing 5 g of dried plant tissue into a pre-dried and tared cubicle. The cubicles were placed into a pre-heated oven at 103 °C for 24 h, after which they were allowed to cool in a desiccator and re-measured. The results were expressed as a percentage of dry matter:

$$TH\%=\left(\frac{M_{0}-M_{1}}{M_{0}}\right)\times 100$$

where

1. TH%—humidity level;
2. M_0_—initial mass before drying;
3. M_1_—mass obtained after drying.

#### 4.4.2. pH Determination

A total of 2 g plant tissue was placed in 10 mL of hot double-distilled water, H_2_O, after which the solution was filtered and allowed to cool. The pH of the filtrate was measured by immersing an electrode of a pH meter. 

#### 4.4.3. Ash and Organic Matter Content

The calcination procedure of the plant material was based on the guidelines of the French association of standardization standard (ISO 928:1997) [55]. The samples were placed in a furnace at a temperature of 550 °C until white ashes of a constant mass were obtained. The organic matter was expressed as a percentage using the formula below:

$$OM\%=\left(\frac{W_{0}-W_{1}}{WS}\right)\times 100$$

where

OM%—organic matter;
W_0_—initial mass of the capsule before calcination;
W_1_—mass of the capsule after calcination;
WS—mass of the sample.

After the determination of the organic matter content, the ash content was calculated based on the following equation:

Ash%=100−OM%

#### 4.4.4. Determination of Mineral Contents

The mineral content was determined as described in Miller et al. [56].

Briefly, 0.25g of homogenized and lyophilized *M. friwaldszkyana* plant material was weighed into TFM Multiwave 3000 vessels (Anton Paar, Santa Clara, CA, USA). Concentrated HNO_3_ (trace metal grade) and 2 mL of 30% H_2_O_2_ were added to each vessel, followed by 30 min incubation before processing. 

ICP Multi-element Standard Solution IV Certipur^®^ (Merck) was used as a calibration standard, and the measurements of the analytes were performed on a 7850 ICP-MS device (Agilent Technologies, Santa Clara, CA, USA). The system’s parameters were set as follows: RF power: 1600 W, plasma argon flow rate: 15.0 L min^−1^, nebulizer gas flow rate: 0.9 L min^−1^.

### 4.5. Data Analysis

The data shown are based on six biological replicates. R version 4.3.0 (R Core Team, 2023) was applied for the analyses of primary and secondary metabolites. The _ggplot2_package [57] was used for the preparation of the boxplots. The top 20 most abundant metabolites were determined and ordered based on their mean relative abundance.

Each of the lipid groups was calculated as a percentage of the total lipid content, and the presented data are the means of six biological replicates.

The lipidomics data were imported into Microsoft Excel, and each of the detected lipid groups was calculated as a percentage of the total lipid content. The data were presented as the means of six biological replicates. 

### 4.6. Animals

Male Wistar rats with an average weight of 150–270 g were used. The animals were housed under the following standard laboratory conditions: temperature: 22 °C ± 1 °C, humidity: 45%, 12:12 h light/dark cycle, and food and water ad libitum. 

### 4.7. Acute Toxicity

The methanolic extract was obtained, and the solvent (methanol) was evaporated, as described in Section 4.1. The dried extract was dissolved in water until the corresponding concentrations required for the evaluation of acute toxicity as well as the anti-inflammatory effects were obtained.

Acute toxicity testing was performed as described in the scientific literature [58] with some modifications. Briefly, eight groups of three animals (160–270 g) were treated orally with a water solution of the desiccated methanolic extract in doses of 5000, 2000, 1500, 1000, 800, 600, 400, and 200 mg/kg. Substantial observation was conducted for 24 h, with a focus on lethality and signs of toxicity. At the 24th hour, the mortality in each group was checked. The LD50 value was calculated by the following formula:

LD50 = (M_0_ + M_1_)/2

- M_0_—the highest dose leading to no mortality among treated animals;
- M_1_—the lowest dose at which mortality is detected among treated animals.

The number of the rats was chosen according to the current principle of the 3R for the humane treatment of laboratory animals (Replacement, Reduction and Refinement). In order to decrease the number of animals used in the experiments, we employed groups of 3 in accordance with a recent research paper [58].

### 4.8. Carrageenan-Induced Paw Edema

The experiment was performed as described previously [59] with slight modifications. Male Wistar rats (weight 150–180 g) were divided into six groups (*n* = 8) and treated as follows: controls received saline (0.1 mL/100 g bw); positive controls (diclofenac) received diclofenac sodium in a dose of 25 mg/kg bw; and experimental groups 3, 4, and 5 received 250, 400, and 500 mg/kg bw dried methanolic extract of *M. frivaldszkyana* dissolved in water, respectively; group 6 received a solution of RA in saline at a dose of 30 mg/kg. The referent substance RA was obtained from Sigma. The saline, *M. frivaldszkyana* extracts, and RA were applied orally for 14 days using a gastral tube, while diclofenac was given once on the day of the experiment via the same route. Rat paw edema was induced one hour after the last treatment. A one-percent solution of λ-carrageenan in saline (0.1 mL) was injected subplantarly into the right hind paw. A plethysmometer apparatus (Ugo Basile, Gemonio, Italy) was employed to measure the paw volume before the injection (*V*_0_) and at the 1st, 2nd, 3rd, 4th, and 5th hours after the injection (*Vn*).

The percentage of the increase in the paw volume was evaluated according to the following formula:

Percentage of increase (%) = [(*Vn* − *V*_0_)/*V*_0_] × 100

### 4.9. Statistical Analysis

The results from the in vivo experiments were analyzed with the software SPSS 17.0. The one-sample Kolmogorov–Smirnov test showed a normal distribution of the data. One-way ANOVA and Bonferroni’s post hoc test were used for further evaluation. The results are presented as means ± SEM and were considered significant at *p* < 0.05. The number of tested animals is given as *n*.

## 5. Conclusions

The complete analysis of *M. frivaldszkyana* methanolic extract revealed sucrose, glucose, mannose, fructose, polyphenols, and sugar alcohols as the primary metabolites in the highest amounts. Triacylglycerols were the most abundant lipid group. The inorganic elements K, Mg, Zn, and Ca were also present in high amounts. Regarding the second metabolites, flavonoids and polyphenols were identified as the most abundant. The extract was particularly rich in linarin, chlorogenic and rosmarinic acid, rutin, eupatorin, kaempferol, and apigenin. The in vivo evaluation of the acute toxicity showed no mortality and no toxic effects in male Wistar rats after oral application in doses of up to 5000 mg/kg bw. Additionally, a 14-day oral application of 250, 400, and 500 mg/kg bw of *M. frivaldszkyana* extract revealed its anti-inflammatory potential in a model of carrageenan-induced paw edema in rats. The anti-phlogistic effect was most present at the 1st, 2nd, and 3rd hours of the inflammation. This activity may be related to the high concentration of flavonoids in the extract, as the literature overview revealed the anti-inflammatory properties of these compounds. Based on these results, we propose that the high content of flavonoids (chlorogenic and rosmarinic acid, rutin, eupatorin, kaempferol, and apigenin) is probably the main factor related to the observed anti-inflammatory activity of the extract.

This study has the following limitations: (1) it was performed on rats after 14 days of oral intake, and different treatment periods can induce different outcomes; (2) results obtained in rats cannot be extrapolated to humans without additional experiments; (3) the acute toxicity study was performed on animals, and no primary investigation on cell lines was carried out, so it is difficult to obtain a more detailed view of the molecular pathways involved in the anti-inflammatory activity; and (4) the total plant extract was applied in this study, and the results suggest a synergetic action of the important plant metabolites; however, it is challenging to understand which of them are the most important players in the immune response.

Given the high content of linarin in the obtained extract, we suggest some beneficial effects on liver function. The object of our future exploration will be the effect of the extract on animal models of liver toxicity.

## Figures and Tables

**Table 1:** Humidity level (TH), pH, and organic matter (OM) and ash content of the used *M. frivaldszkyana* plant material.

TH (%)	pH	Ashes (%)	OM (%)
19.4 ± 0.92	5.853 ± 0.032	88.28 ± 0.25	11.72 ± 0.043

**Table 2:** Concentrations (mg/kg) of elements in analyzed *Micromeria* samples. Average values (*n* = 3) with relative standard deviation (RSD) in brackets.

**Ca**	**K**	**Mg**	**Na**	**B**	**Al**
283.7 (3.2)	8734.6 (4.4)	2094.3 (3.5)	11.5 (4.4)	5.4 (3.5)	28.5 (2.7)
**Mn**	**Fe**	**Cu**	**Zn**	**Sr**	**Ba**
17.6 (3.0)	67.1 (4.0)	2.5 (1.8)	534.6 (0.3)	6.1 (2.7)	6.0 (1.2)

**Table 3:** Acute toxicity test of methanolic extract of *M. frivaldszkyana*.

Dose (mg/kg bw)	200	400	600	800	1000	1500	2000	5000
Mortality	0/3	0/3	0/3	0/3	0/3	0/3	0/3	0/3
Toxic effects	None observed	None observed	None observed	None observed	None observed	None observed	None observed	None observed

## Data Availability

The data presented in this study are available on request from the corresponding author.

## Supplementary Materials

The following supporting information can be downloaded at: https://www.mdpi.com/article/10.3390/ijms25105396/s1.
